# The Level of Vision Necessary for Competitive Performance in Rifle Shooting: Setting the Standards for Paralympic Shooting with Vision Impairment

**DOI:** 10.3389/fpsyg.2016.01731

**Published:** 2016-11-08

**Authors:** Peter M. Allen, Keziah Latham, David L. Mann, Rianne H. J. C. Ravensbergen, Joy Myint

**Affiliations:** ^1^Department of Vision and Hearing Sciences, Anglia Ruskin UniversityCambridge, UK; ^2^Vision and Eye Research Unit, Anglia Ruskin UniversityCambridge, UK; ^3^Research Institute MOVE Amsterdam, Department of Human Movement Sciences, Vrije Universiteit AmsterdamAmsterdam, Netherlands; ^4^Postgraduate Medicine, Life and Medical Sciences, University of HertfordshireHatfield, UK

**Keywords:** visual acuity, contrast sensitivity, vision impairment, shooting, classification, para-sport

## Abstract

The aim of this study was to investigate the level of vision impairment (VI) that would reduce performance in shooting; to guide development of entry criteria to visually impaired (VI) shooting. Nineteen international-level shooters without VI took part in the study. Participants shot an air rifle, while standing, toward a regulation target placed at the end of a 10 m shooting range. Cambridge simulation glasses were used to simulate six different levels of VI. Visual acuity (VA) and contrast sensitivity (CS) were assessed along with shooting performance in each of seven conditions of simulated impairment and compared to that with habitual vision. Shooting performance was evaluated by calculating each individual’s average score in every level of simulated VI and normalizing this score by expressing it as a percentage of the baseline performance achieved with habitual vision. Receiver Operating Characteristic curves were constructed to evaluate the ability of different VA and CS cut-off criteria to appropriately classify these athletes as achieving ‘expected’ or ‘below expected’ shooting results based on their performance with different levels of VA and CS. Shooting performance remained relatively unaffected by mild decreases in VA and CS, but quickly deteriorated with more moderate losses. The ability of visual function measurements to classify shooting performance was good, with 78% of performances appropriately classified using a cut-off of 0.53 logMAR and 74% appropriately classified using a cut-off of 0.83 logCS. The current inclusion criteria for VI shooting (1.0 logMAR) is conservative, maximizing the chance of including only those with an impairment that does impact performance, but potentially excluding some who do have a genuine impairment in the sport. A lower level of impairment would include more athletes who do have a genuine impairment but would potentially include those who do not actually have an impairment that impacts performance in the sport. An impairment to CS could impact performance in the sport and might be considered in determining eligibility to take part in VI competition.

## Introduction

Paralympic athletes undergo *classification* to determine whether they should be eligible to compete in the sporting event, and to ensure that they compete against other athletes who have a similar level of impairment (International Paralympic Committee, 2007, The *Classification Code* of the IPC explicitly details the need for the development and implementation of robust classification systems that are evidence-based and sport-specific (Tweedy and Vanlandewijck, 2011; Tweedy et al., 2014). Although this process has for some time been underway for athletes with physical or intellectual impairments, at this stage there has been minimal change to the classification systems for athletes with VI (Ravensbergen et al., 2016). For VI sport, the current classification system requires athletes to be tested on a maximum of two different elements of visual function: distance VA and VF, with eligible athletes classified to compete in one of the B3, B2, or B1 classes (from lowest to highest impairment) depending on their level of impairment. These criteria were originally based on the World Health Organization’s criteria for low vision and blindness (World Health Organization, 2004), and as a result there is no evidence to show that the classes reliably represent categories of impairment that have distinct effects on sport performance.

Shooting is a particularly appealing sport for people with VI, because competitors are allowed, in the *adapted* form of the sport, to use an audio signal to help them guide the direction of the gun barrel toward the target. We have recently shown (Myint et al., 2016) that athletes with VI are successfully able to use that auditory information to compensate for their impairment and enhance performance in rifle shooting, with the findings showing that athletes with complete blindness perform no worse than those athletes with residual vision when auditory guidance is available. This finding would suggest that all athletes competing in VI shooting should participate within the same class irrespective of their level of impairment because athletes with complete blindness can compete fairly against, and are at no performance disadvantage, when competing against athletes with some residual vision.

Although the existing evidence points toward the need for only one class during competition for VI shooting, what remains unknown is what might be an appropriate *minimum level of impairment* required for a person to qualify to compete in competition. The current rules for classification require an athlete to have a VA worse than 1.0 logMAR (6/60 or 20/200), or a VF of less than 20 degrees radius (International Paralympic Committee, 2007). However, because those criteria were arbitrarily set on the basis of the criteria for legal blindness originally established by the World Health Organization (2004), it is unclear whether these eligibility criteria would represent a level of impairment that would significantly impact performance in shooting. If the current inclusion criteria represent a level of impairment that does not actually impact shooting performance, then this would mean that there may be some athletes presently competing who do not have a disadvantage in the sport and therefore should not qualify to compete. Conversely, the current level of impairment could be too high, meaning that some people who do have a genuine visual disadvantage when competing would be presently ineligible to compete in the sport. Moreover, the current system of classification accounts for only VA and VF, and it could be that there are other aspects of vision (for instance CS) that could adversely influence performance yet are not accounted for in the present system of classification (Ravensbergen et al., 2016).

The minimum level of impairment required to compete in VI sport should be the level of impairment that has an impact on sport performance in the *unadapted* rather than the adapted form of the sport. That is, the level of impairment that would decrease performance in the absence of any adaptation put in place to benefit athletes with VI, in this case in the absence of auditory guidance. The reason for this is that the sport should cater for people who are at a disadvantage when competing against other athletes who do not have VI. To illustrate this point, it would not be possible to set a minimum impairment criteria on the basis of the adapted form of shooting (with auditory guidance) because even people who are completely blind do not perform worse than people with moderate levels of vision (1.0 logMAR) (Myint et al., 2016) and therefore there appears to be no level of VI at which performance is decreased. An evidence-based minimum impairment criterion should ensure that athletes who are disadvantaged as a result of their impairment in the *unadapted* form of the sport are eligible to compete in the *adapted* form of the sport, and will ensure that they compete only against others who have an impairment that does impact performance.

The aim of this study was to establish the level of VI that would reduce performance in shooting. The shooting performance of international-level shooters without VI was assessed in the *unadapted* form of the sport while wearing lenses that simulated a range of different levels of VI. The results were expected to demonstrate the level of vision necessary for competitive performance in shooting, and in the process would provide guidance for an appropriate minimum impairment criterion to be used for Paralympic shooting for athletes with VI.

## Materials and Methods

### Participants

Nineteen elite able-sighted shooters (nine male, all competing at international level at the time of testing) took part in the study (*M*_age_ ±*SD* = 21.0 ± 6.8; range 17–45 years). Participation in the study was voluntary, with all athletes agreeing to participate without reward or incentive. The Faculty Research Ethics Panel at Anglia Ruskin University, Cambridge, UK, gave ethical approval for the study. All participants provided informed consent and the research was conducted in accordance with the tenets of the Declaration of Helsinki.

### Procedure

All data were collected during National training camps for either the English (at the West Midlands Regional Shooting Center) or the Welsh shooting squads (at the Sports Wales National Center). The shooting ranges were equivalent, both being 10 m indoor rifle ranges with standardized lighting at the target of a minimum of 1500 lux and a maximum of 1800 lux (at both ranges). Vision and shooting performance were assessed in each of seven different vision conditions (habitual vision + six levels of simulated VI).

#### Measurement of Vision

For each of the seven vision conditions, two tests of visual function were performed on the shooting eye under standardized lighting conditions (measured as ≈ 200 lux or ≈ 32–64 cd/m^2^). This lighting level is slightly less than what is recommended when testing VA (80–320 cd/m^2^; British Standards Institution, 2009) but was uniform across both testing locations. The tests were chosen as they were assumed to be tests that measured visual parameters likely to be related to performance in shooting. First, a test of *distance* VA was performed using an externally illuminated ETDRS LogMAR letter chart at 4 m (2000 Series Revised, Precision Vision, La Salle, IL, USA). Letter by letter scoring was used with the acuity measured in logMAR units. On the logMAR scale, smaller logMAR scores indicate better VA. Although a tumbling E logMAR chart is used currently for the purposes of classification, the ETDRS logMAR chart produces very similar levels of acuity (Bourne et al., 2003). Second, a test of CS was performed using a Pelli-Robson chart (Pelli et al., 1988) at 1 m. A letter by letter scoring method was used for the habitual vision and a triplet scoring method (two out of three correct identifications) for CS measures under simulated VI (Elliott et al., 1991). Higher logCS scores indicate better CS (testing range 0–2.25 logCS).

#### Simulated Vision Impairment

Cambridge simulation glasses (*sim-specs*) were used to simulate increasing levels of VI (Goodman-Deane et al., 2013). The sim-specs consist of diffusing filters that block and scatter light to reduce visual performance, and are mounted in cardboard frames so that both eyes look through separate filters. The filters can be used singly or in combination to provide progressive increases in simulated impairment. We used one to six filters in front of each eye to simulate six different levels of VI (termed ‘Level 1’ to ‘Level 6’). The sim-specs result in decreases in both VA and CS (Rae et al., 2015) and so both measures were assessed in each level of simulated impairment to examine the combined effect of decreases in VA and CS on shooting performance. Shooters can also qualify to compete in VI shooting on the basis of impairment to their VF, however, we did not attempt to simulate an impaired VF in this particular experiment.

#### Effect of Simulated Vision Impairment on Shooting Performance

Participants were required to shoot a regulation competition air rifle, while standing, toward a regulation target placed at the end of a 10 m shooting range. Scoring was performed using an electronic scoring system (SCATT) rather than through the use of actual pellets. The target replicated that used during competition, consisting of ten rings so that there was a central circle surrounded by nine concentric annuli, with the athlete scoring 10 for a hit in the central circle, 9 for the immediately surrounding annulus, 8 for the next annulus, and so on. Although any further subdivisions were not visible to the participants, the SCATT scoring system further subdivided each of the ten rings into 10 concentric annular ‘score zones’ of equal width with increments of 0.1 between zones. As a result, the highest score for an individual shot was 10.9.

Participants took part in a 5-min warm up before commencing the experiment proper. Three shots were taken toward the target in each of the seven vision conditions and these were used for the analyses. We chose to include only a small number of shots in each condition because this is more representative of the demands of competition where international athletes must perform at a high level on every shot. Participants first shot with their habitual vision (no sim-specs in place) before shooting in each of the six simulated impairment conditions that were presented in a randomized order for each participant.

#### Analysis

In order to set a shooting performance threshold below which, we would consider shooting performance to be below habitual performance, we first defined the *normalized shot score* for each individual shot in the habitual condition. To calculate this, we normalized each individual’s shot by that person’s mean across their three shots in the habitual condition $\left(i.e.\,,\;Normalized\,\;shot\,\;score=\frac{Individual\,\;\;shot\,\;\;\,score}{Three\,\;shot\,\;average}\times 100\right).$ The lower boundary of the 99% confidence interval around the mean was then set as the cut-off point below which performance would be below the expected level of habitual performance.

In order to determine the level of VA and CS that would result in a significant reduction in shooting performance, ROC curves were constructed to examine how well VA and CS cut-off levels could classify the athletes as having achieved an ‘expected’ or ‘below expected’ level of shooting performance. The ROC curve considers both the *sensitivity* and the *specificity* of any chosen cut-off. Sensitivity quantifies how well the vision criterion identifies athletes whose shooting performance is poorer than expected. Specificity represents the ability to appropriately identify those with shooting scores that are as expected when using that vision cut-off point. The ability of a test to act as a suitable discriminator of performance can be evaluated using the AUC of the ROC curve. A test with perfect discrimination will have an AUC of 1.0, and one with no discriminative ability will have an AUC of 0.5.

A further parameter of interest is Youden’s J, which provides a measure of the proportion of the entire sample who are correctly classified when using a specific cut-off value. Youden’s J = sensitivity – [1-specificity], on a scale of 0–1, where 0 represents no discriminative ability and 1 is perfect discrimination. Youden’s J can be used to define the cut-off that maximizes the proportion of a sample correctly classified, assuming that false positives and false negatives are of equal importance.

## Results

### Habitual Vision

The tests of visual function confirmed that all participants had a good level of habitual visual function (**Table 1**). Habitual VA (-0.09 ± 0.10 logMAR) was consistent with normative values for 18–24 year old adults (-0.13 ± 0.06) (Elliott et al., 1995). Habitual CS (1.96 ± 0.02 logCS) was also consistent with normative values for a Pelli-Robson chart with young adults (1.86 ± 0.09 logCS) (Elliott and Bullimore, 1993).

**Table 1:** Visual function of the 19 international-level shooting athletes.

	VA (logMAR)				CS (logCS)			
	Mean	*SD*	Min	Max	Mean	*SD*	Min	Max
Habitual	-0.09	0.10	-0.30	0.12	1.96	0.02	1.90	2.00
Level 1	0.04	0.10	-0.10	0.22	1.59	0.10	1.35	1.80
Level 2	0.23	0.16	0.04	0.74	1.30	0.10	1.05	1.50
Level 3	0.46	0.14	0.24	0.88	1.01	0.15	0.60	1.35
Level 4	0.73	0.14	0.50	0.92	0.73	0.13	0.45	1.05
Level 5	0.98	0.12	0.74	1.20	0.45	0.17	0.00	0.75
Level 6	1.29	0.14	1.00	1.60	0.13	0.19	0.00	0.45

### Habitual Shooting Performance

The mean of the three shots taken in the habitual condition for each individual was a score of 9.8 ± 0.4 (range 9.1 – 10.3; 99% CI = 9.6 – 10.1), demonstrating the high level of performance in our participants.

The individual normalized shot scores across all participants were 100.0 ± 5.0% (Mean ± SD) with a 99% confidence interval from 87.0 to 113.0%. The cut-off point below which performance would be ‘below expected’ was therefore 87%.

### Visual Performance with Simulated Vision Impairment

Consistent with previous reports (Rae et al., 2015), the progressive increase in simulated impairment produced by the sim-specs resulted in linear reductions in both VA and CS. Each progressive increase in filter level resulted in a mean reduction of 0.23 logMAR units for VA (linear regression equation: VA = -0.18 +(0.23 × filter level); *R*^2^ = 0.96) and 0.30 log units for CS (linear regression: CS = 1.91 + (-0.30 × filter level); *R*^2^ = 0.98), with the progressive increase in filter level reducing VA and CS to a similar degree (VA = 1.30-(0.76 × CS); *R*^2^ = 0.90).

### Shooting Performance with Simulated Vision Impairment

Normalized shooting performance in each of the six levels of simulated VI was determined by calculating each individual’s mean score across their three shots in that condition, and normalizing that score by expressing it as a percentage of the baseline performance achieved with habitual vision.

**FIGURE 1:**
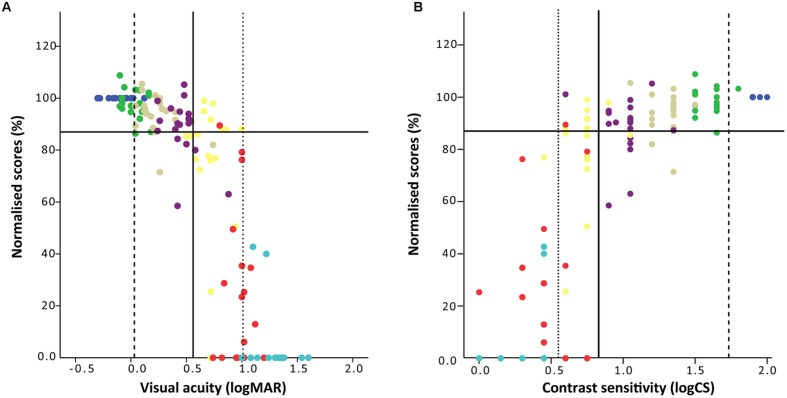
**Shooting performance as a function of (A) VA and (B) CS.** The *x*-axes show the level of visual function achieved by each of the 19 participants in each of the seven levels of simulated VI (habitual vision – violet, filter level 1 – indigo, 2 – blue, 3 – green, 4 – yellow, 5 – orange, and 6 – red), with lower logMAR values representing better VA, and higher logCS values representing better CS. The *y*-axis shows the shooting performance as a percentage of the habitual baseline performance. The solid horizontal lines in **(A,B)** indicate the cut off point below which shooting performance is considered to be significantly worse than baseline (determined by the lower bound of the 99% confidence interval at baseline, in this case 87% of the baseline score). The solid vertical line represents the Youden’s J, the dashed vertical line maximum sensitivity and the dotted vertical line maximum specificity.

A non-linear relationship was found to exist between VA and the normalized score for the three shots taken in each of the simulated VI conditions (**Figure 1A**). Shooting performance remained relatively unchanged in response to mild reductions in VA (i.e., low levels of simulated impairment) but then deteriorated more quickly with further reductions in acuity. A similar non-linear relationship was found between CS and normalized shooting score (**Figure 1B**), with more marked decreases in shooting performance apparent only with relatively higher reductions in CS.

**FIGURE 2:**
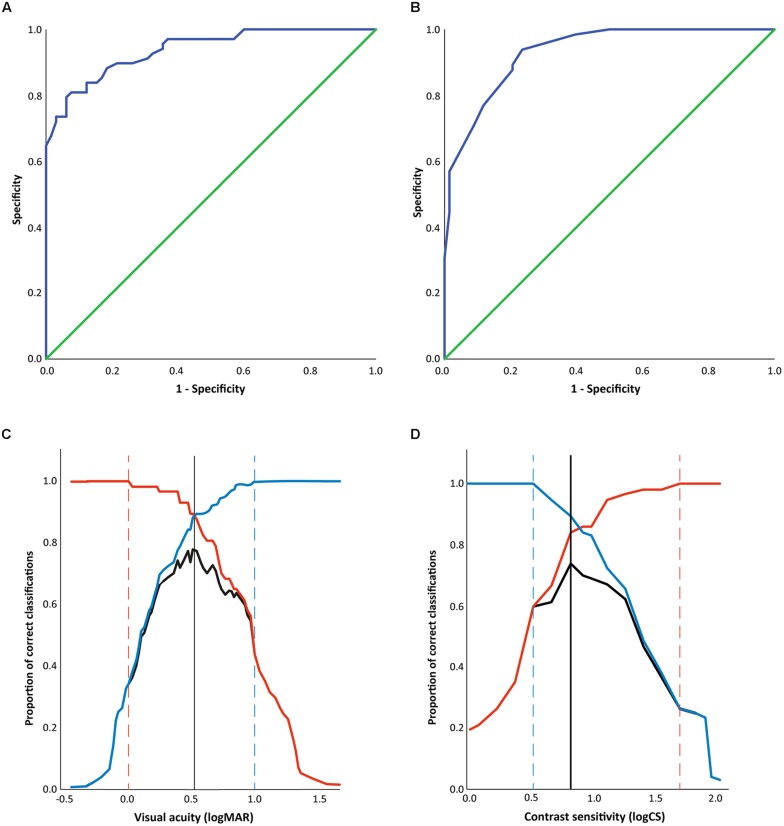
**(A,B)** Receiver Operating Characteristic curves showing the sensitivity and specificity of **(A)** distance VA cut-off and **(B)** CS cut-off in correctly classifying athletes as achieving ‘expected’ or ‘below-expected’ performance. The diagonal green reference line represents an AUC of 0.5, indicating that a test would have no discriminative ability to classify athletes. Perfect discrimination of athletes’ performance by visual function (AUC = 1.0) would be achieved with a line following the top left hand corner of the graph. The blue line indicates the performance of the visual functions, with both distance VA **(A)** and CS **(B)** having an AUC of 0.94 ± 0.02 (*p* < 0.01 to reject the null hypothesis that the AUC is 0.5 in both cases). **(C,D)** Sensitivity (red), specificity (blue) and Youden’s J (black) for cut offs of different levels of **(A)** VA, and **(B)** CS. Sensitivity indicates the proportion (0–1) of those with below expected shooting scores who have visual function poorer than the cut-off, and the dashed red line indicates the cut off providing maximum sensitivity. Specificity indicates the proportion (0–1) of those with expected shooting scores who have visual function better than the cut-off value, and the dashed blue line indicates the cut off providing maximum specificity. Youden’s J indicates the proportion of the entire sample correctly classified by the cut off, with the maximum value indicated by the black line.

Receiver Operating Characteristic curves for VA (**Figure 2A**) and CS (**Figure 2B**) demonstrate how well cut-off levels classify athletes as having achieved an ‘expected’ or ‘below expected’ level of shooting performance. For VA, the AUC was 0.94 ± 0.02 (*p* < 0.001 to reject the null hypothesis that the AUC is 0.5; 95% confidence interval for the AUC = 0.91 – 0.98), demonstrating that it has high discriminative ability to differentiate between those with above and below expected levels of performance. From the ROC analysis, the performance of the different cut off values can be compared (**Figure 2C**). Maximum sensitivity is achieved with a VA of 0.03 logMAR, though at this point specificity is poor as many individuals with an expected level of performance would be included. Maximum specificity is at 1.00 logMAR, though this results in poor sensitivity, with a large proportion of shooters with below expected levels of performance being excluded. A maximal Youden’s J value of 0.78 occurs at 0.53 logMAR, indicating that this cut-off correctly classifies 78% of shooters.

The ROC curve for CS indicates similarly good discrimination between shooters with expected and below expected levels of performance (AUC = 0.94 ± 0.02; *p* < 0.001, 95% confidence interval = 0.90–0.98). The cut-off level of CS that would achieve maximum sensitivity is 1.73 logCS, for maximum specificity is 0.53 logCS, and for the maximum Youden’s J (0.74) is at 0.83 logCS (**Figure 2D**).

## Discussion

The aim of this study was to establish the level of VI that would reduce performance in shooting. We simulated six different levels of VI in 19 international-level shooters who would usually compete without VI, and investigated the impact of the simulated impairment (in terms of simultaneous reductions in VA and CS) on their shooting performance. The results revealed that mild reductions in VA and CS had very little impact on performance, demonstrating that optimal vision might not be required for competitive performance in the sport. Crucially, more moderate reductions in both acuity and CS (poorer than 0.5 logMAR and 0.8 logCS) were associated with poorer shooting performance, demonstrating the level of impairment that produces a significant decrease in shooting performance. The results demonstrate the level of vision required for competitive performance in shooting. Further work is required to determine the impact on shooting performance of *independent* reductions in VA and CS in order to determine the minimum impairment criteria required in Paralympic shooting for athletes with VI.

Performance in visually guided motor actions (such as those produced in many sports) has often been shown to be surprisingly unaffected by below-normal levels of vision. For instance, very high levels of simulated vision loss are required to reduce performance in static aiming tasks such as a basketball free-throw (1.9 logMAR; 6/450 or 20/1500) (Applegate and Applegate, 1992; Bulson et al., 2015) and in golf putting (also 1.9 logMAR) (Bulson et al., 2008). Further, high levels of impairment are required to decrease performance even when performing some of the most challenging interceptive sporting tasks, for example when hitting a fast-moving cricket ball (0.5–0.8 logMAR; 6/20–6/49 or 20/67–20/160) (Mann et al., 2007, 2010a,b). In this study, we have again shown the resilience of a visually guided action to below normal levels of vision, with moderate levels of impairment required to reliably reduce performance. What is clear in comparison to other studies though is that the level of impairment necessary to reduce performance in shooting is much less than that required in other static aiming tasks (such as basketball and golf), and is comparable to the level required in the more dynamic task of cricket batting. This finding confirms the visually demanding nature of shooting (Erickson, 2007).

From a Paralympic eligibility standpoint, it is important to establish a reliable and valid cut-off level of vision below which a person would be considered eligible to compete in the sport as an athlete with VI. This cut-off point should be the level of VI that clearly reduces performance in the *un-adapted* form of the sport. An ideal classification system will maximize the inclusion of athletes who *do* have an impairment that legitimately impacts their performance in the sport, and therefore would be appropriately classified as being eligible to compete in Paralympic competition (i.e., it would have high *sensitivity*). A system with poor sensitivity represents a substantial problem, because it would exclude athletes whose vision does impact their performance in the sport. To ensure the integrity of the competition the system must also minimize the inclusion of people whose impairment does *not* reduce performance in the sport (i.e., the system must also have high *specificity* to minimize the inclusion of ‘false positives’). Poor specificity represents a significant problem because it allows athletes whose vision *does not* impact their performance to compete against others whose impairment does impact their performance. To minimize the chance of this happening, a cut-off that maximizes specificity would be required so that only those with vision that does affect their performance would be included in competition.

It is highly unlikely that there will be any test criterion or cut-off point that perfectly identifies individuals whose visual status reduces their sporting performance. An ideal test will maximize the proportion of people appropriately categorized, and it should consider whether it is more important to prioritize sensitivity or specificity. This is a decision that must be made by the governing body of the sport. If sensitivity and specificity were to be considered equally important in Paralympic sport then our findings show that a VA of 0.53 logMAR (Snellen equivalent 6/20 or 20/68) with a CS of 0.83 logCS would maximize the proportion of the potential athletes appropriately classified as eligible to compete. If considered by themselves, either of these VA or CS cut-offs would provide both high sensitivity and specificity (≈0.8-0.9) and correctly classify approximately 75% of all athletes. If, however, one of sensitivity or specificity is considered to be relatively more important than the other, then alternative criteria maximizing categorisation accuracy under these conditions is required.

A highly inclusive cut-off point would be one that maximized the inclusion of people whose performance in the sport was genuinely impacted by their VI. While being inclusive, such a system would increase the likelihood of people being included whose performance is not impacted by their level of vision (i.e., the system would have high sensitivity yet poor specificity). If a very inclusive approach were to be desirable, then our results show that maximum sensitivity would be achieved at a VA of 0.03 logMAR (6/6.5 or 20/21.4) and a CS of 1.7. Whilst sensitive (1.00), this value is clearly not specific (0.34) as it would also allow access to vision impaired shooting for many people who would not be considered to be VI, and more importantly for whose vision would allow them to achieve their optimal level of performance in the sighted form of the sport. A point worthy of consideration though is that the influence of a criterion which allowed the inclusion of people whose vision did not impact performance in the sport would depend on whether residual vision provided an advantage in the adapted form of the sport. In the case of shooting where the adapted format allows athletes to use auditory guidance to guide the direction of the gun barrel, it appears as though those with vision do not hold an advantage over those who are blind, as those with residual vision near the inclusion criterion (1.0 logMAR) perform no better than athletes with no perception of light (Myint et al., 2016). Whether this would still be the case if the VA criterion were moved to a better acuity (or even with unimpaired acuity) is not currently known. However, this might provide some reassurance that a less conservative cut-off criteria would not necessarily disadvantage those with a genuine impairment in the sport.

What is clear from our findings is that the current minimum inclusion criteria for VI shooting (1.0 logMAR) is probably conservative and gives high specificity, ensuring that those whose impairment does not impact performance are not included in competition. Our findings for VA show that specificity is maximized at 1.0 logMAR (actually at maximum specificity of 1.0; **Figure 2C** and dashed vertical line in **Figure 1A**), and would maximize the chance that only those with VI that does impact performance in sighted shooting would be included for competition in VI shooting. The potential drawback of this cut-off point though is the poor sensitivity in identifying those whose shooting performance is impaired by their vision (sensitivity = 0.44), with a considerable number of people being excluded from competition whose impairment probably *does* impact their performance in the *unadapted* form of the sport.

If CS is considered as an additional or alternative means of assessing the cut-off criterion for VI shooting, then more information might be required on its impact on performance independent to a reduction in VA. In this study, we used sim-specs that reduced VA and CS in a similar fashion. Whilst similar reductions in both VA and CS are likely in many ocular pathologies such as cataract (Elliott and Hurst, 1990; Elliott and Situ, 1998), the simulated visual impairment assessed here may not reflect findings with VI athletes who have a preferential loss of either their VA or CS alone. Further work is required to establish the independent impact of losses of VA and CS on shooting performance to determine whether athletes should be eligible to compete on the basis of an independent reduction in one of those two visual parameters. It could be that the reductions in performance seen here were a result of the decrease in VA and were not impacted by the changes in CS (or vice-versa). Alternately, if the decrease in performance was a *combined* result of the decreases in acuity and CS then the actual level of VA (or CS) alone that decreases performance would be expected to be worse (i.e., a higher logMAR value) than that found here. Further work is required that, if possible, more independently simulates decreases in VA and CS to provide a clearer understanding of what should be the minimum decrease in visual function necessary to decrease shooting performance, and therefore to inform the minimum impairment criteria for Paralympic competition. Moreover, additional work is required to determine what should be the minimum VF that would impact performance in the *unadapted* form of the sport and therefore qualify a person to compete in VI shooting.

## Conclusion

The level of VI necessary to compete in VI shooting should be the level that impacts performance in the *unadapted* form of the sport. We have found that mild reductions in both distance VA and CS have no adverse effect on shooting performance, and that more moderate reductions in VA and CS occurring together are required to decrease performance. The current system of classification for VI shooting requires shooters to have a distance VA of 1.0 logMAR at best, and our results show that this cut-off would maximize the chance of including only those with vision that would impair their ability to shoot in sighted competition, assuming that both VA and CS were affected. If greater sensitivity is required to include more of those whose performance is impaired by their vision, then a better VA would provide a more appropriate cut-off level. Results suggest that the relevance of impaired CS should be considered in determining eligibility to shoot in VI competition.

## Author Contributions

All authors conceived and designed the study. PA and JM collected the data, PA, KL, DM, and RR analyzed the data. All authors wrote, reviewed, edited, and approved the manuscript.

## Conflict of Interest Statement

The authors declare that the research was conducted in the absence of any commercial or financial relationships that could be construed as a potential conflict of interest.

## Abbreviations

AUC: area under the curve
CS: contrast sensitivity
ETDRS: early treatment diabetic retinopathy study
IPC: international paralympic committee
ROC: receiver operating characteristic
VA: distance visual acuity
VF: visual field
VI: visually impaired or vision impairment.
